# Exploring the potential role of *GyrA* inhibiting quinoline analog: an in silico study

**DOI:** 10.1038/s41598-025-04409-2

**Published:** 2025-07-02

**Authors:** Soumyadip Ghosh, Sudha Ramaiah

**Affiliations:** 1 Medical and Biological Computing Laboratory, School of Biosciences and Technology (SBST), Vellore Institute of Technology (VIT), Vellore, India; 2 Department of Bio Sciences, SBST, VIT, Vellore, 632014 India

**Keywords:** AMR, Mutations, *Pseudomonas aeruginosa*, Fluoroquinolone, Ciprofloxacin, Levofloxacin, Antimicrobials, Bacteriology, Pathogens, Computational biology and bioinformatics, Drug discovery, Structural biology

## Abstract

Fluoroquinolone-resistant *Pseudomonas aeruginosa* poses a significant global health concern, particularly in healthcare settings. This opportunistic pathogen has developed resistance against multiple classes of antibiotics, rendering infections challenging to treat. The present study focused on identifying quinoline analogs as potential inhibitors of gyrA in fluoroquinolone-resistant *P. aeruginosa*. Utilizing structural bioinformatics, molecular docking, molecular dynamics (MD) simulations, and MM/PBSA binding energy analyses, the quinoline analog, N-benzylquinoline-8-sulfonamide (M2), emerged as the most promising candidate. Molecular docking revealed M2’s better binding affinity to gyrA wild type as well as frequently observed mutants, demonstrated average binding energy of − 8.14 kcal/mol, significantly better than ciprofloxacin (− 7.13 kcal/mol) and levofloxacin (− 6.57 kcal/mol). M2 exhibited a robust inhibition constant of 1.09 µM, surpassing control antibiotics ciprofloxacin (6.11 µM) and levofloxacin (15.34 µM). MD simulations validated the dynamic stability of M2 and gyrA complexes (wild-type and mutant), whereas MM/PBSA analysis confirmed strong binding energetics. Principal Component Analysis (PCA) further validated the stability of these complexes by identifying the global energy minima across conformational landscapes. M2 exhibited enhanced efficacy and stability against resistance-associated mutations compared to the standard antibiotics ciprofloxacin and levofloxacin. These findings underscore M2’s potential as a potent therapeutic agent against fluoroquinolone-resistant *P. aeruginosa*. Further experimental validation is necessary to confirm its efficacy and to translate these computational insights into clinical applications.

**Supplementary Information:**

The online version contains supplementary material available at 10.1038/s41598-025-04409-2.

## Introduction

Antimicrobial resistance (AMR) and multidrug resistance (MDR) pose significant threats to global health by compromising the efficacy of current therapies, potentially leading to the pre-antibiotic era. AMR pathogens have already resulted in millions of fatalities, with projections indicating 10 million annual deaths by 2050 if current trends persist^1,2^. Healthcare facilities, once considered safe environments, now harbor MDR bacteria resistant to numerous antibiotics, including fluoroquinolones, thereby limiting the treatment options for critically ill patients^3,4^. Bacteria develop AMR through various mechanisms, such as mutating drug targets to evade antibiotic action^5^. According to recent reports and findings by the WHO, *Pseudomonas aeruginosa (P. aeruginosa)* is a high-risk microorganism that causes high mortality. It is an opportunistic Gram-negative pathogen that causes severe infections, with 40% mortality and substantial morbidity, particularly in cases of pneumonia^6^. Immunocompromised individuals and those with chronic pulmonary diseases such as cystic fibrosis are particularly susceptible to *P. aeruginosa* causing infection. Recent data have indicated an increase in AMR, including that of MDR *P. aeruginosa* strains that employ diverse virulence mechanisms to exacerbate infections^7^. Historically, quinolones, particularly fluoroquinolones, have been effective against *P. aeruginosa*. Ciprofloxacin and levofloxacin are utilized as treatment therapies in numerous cases and remain the sole oral options for quinolone-sensitive *P. aeruginosa*, with ciprofloxacin being superior due to a lower risk of resistance^8,9^. However, resistant strains have limited the efficacy of these antibiotics. Intrinsic resistance mechanisms in *P. aeruginosa* reduce its quinolone sensitivity. The inappropriate use of quinolones has led to acquired resistance, including mutations in DNA gyrase and topoisomerase IV, reduced membrane permeability, and efflux pumps^10,11^. Among these, mutations in gyrA represent the most well-characterized and clinically significant resistance mechanism. Newer fluoroquinolones may be effective; however, resistance can develop. Novel therapies are urgently required to combat MDR *P. aeruginosa*, necessitating a multi-faceted approach.

This study employed structural bioinformatics to identify vulnerabilities in fluoroquinolone-resistant *P. aeruginosa*, focusing on DNA gyrase subunit A (gyrA), a primary target of fluoroquinolone antibiotics, and frequently observed major mutants^12,13^. DNA gyrase, a type II topoisomerase, is an essential bacterial enzyme that introduces negative supercoils into DNA, facilitating DNA replication, transcription and repair^14^. The fluoroquinolones class of antibiotics exert their bactericidal effects by targeting DNA gyrase. These antibiotics bind to quinolone resistance determining region (QRDR) of gyrA, stabilizing DNA-cleaved complexes and preventing bacterial DNA replication^15^. However, mutations within QRDR of gyrA (position 67–106) reduce fluoroquinolone binding affinity, leading to a marked increase in minimum inhibitory concentrations (MICs) and causing fluoroquinolone resistance. Previous studies have demonstrated that single-point mutations (e.g., T83I or D87N) confer significant resistance, whereas double mutations (e.g., T83I_D87N) further enhance resistance, rendering fluoroquinolone ineffective^10^. Considering the widespread occurrence and clinical importance of QRDR mutations in gyrA, our research focused on this as the primary drug target. Our objective is to enhance antimicrobial effectiveness against fluoroquinolone-resistant *P. aeruginosa* by discovering small-molecule inhibitors that demonstrate efficient binding to both wild-type and mutant forms of gyrA. This study proposes a quinoline analog with superior inhibitory effects on gyrA compared to the current antibiotics ciprofloxacin and levofloxacin. The selection of a quinoline analog as a potential therapeutic alternative is based on the structural similarity of quinolines to fluoroquinolones or quinolones and their potential antibacterial activity, which provides a promising foundation for drug development^16,17^. Quinoline and its derivatives have garnered substantial attention in the realm of medicinal chemistry, as evidenced by numerous studies. These compounds have demonstrated a wide array of biological activities, with their antibacterial properties being particularly noteworthy. Research has shown their effectiveness in combating various bacterial pathogens of clinical importance^18–21^. Quinoline is a heterocyclic aromatic organic compound consisting of a fused system of a benzene ring and a pyridine ring^22^. Quinolones are a class of synthetic quinoline-derived antibiotics. Quinolones are structurally derived from quinoline but modified to include functional groups essential for their antimicrobial activity^23^. The shared bicyclic core structure enables quinoline derivatives to effectively target bacterial DNA gyrase and topoisomerase IV, whereas tailored modifications can overcome major resistance mechanisms such as target mutations^24^. The synthetic flexibility of quinoline derivatives facilitates the optimization of their antimicrobial activity, pharmacokinetics, and safety profiles^25^. This characteristic makes quinoline derivatives a rational and strategic choice for developing next-generation therapeutics to combat fluoroquinolone-resistant *P. aeruginosa*.

Cost-effective in silico methods facilitate the discovery of novel drug candidates by identifying critical intermolecular interactions through virtual screening, thereby streamlining the identification of promising leads against fluoroquinolone-resistant *P. aeruginosa*. This study proposes a strategy that utilizes structural bioinformatics to identify potential vulnerabilities in clinically relevant fluoroquinolone-resistant *P. aeruginosa*, and ultimately provides a potential lead candidate. These findings will assist researchers in developing potential therapeutic molecules to combat fluoroquinolone-resistant *P. aeruginosa* infection.

## Results

### Structural retrieval of target modeled proteins and validations

**Fig. 1:**
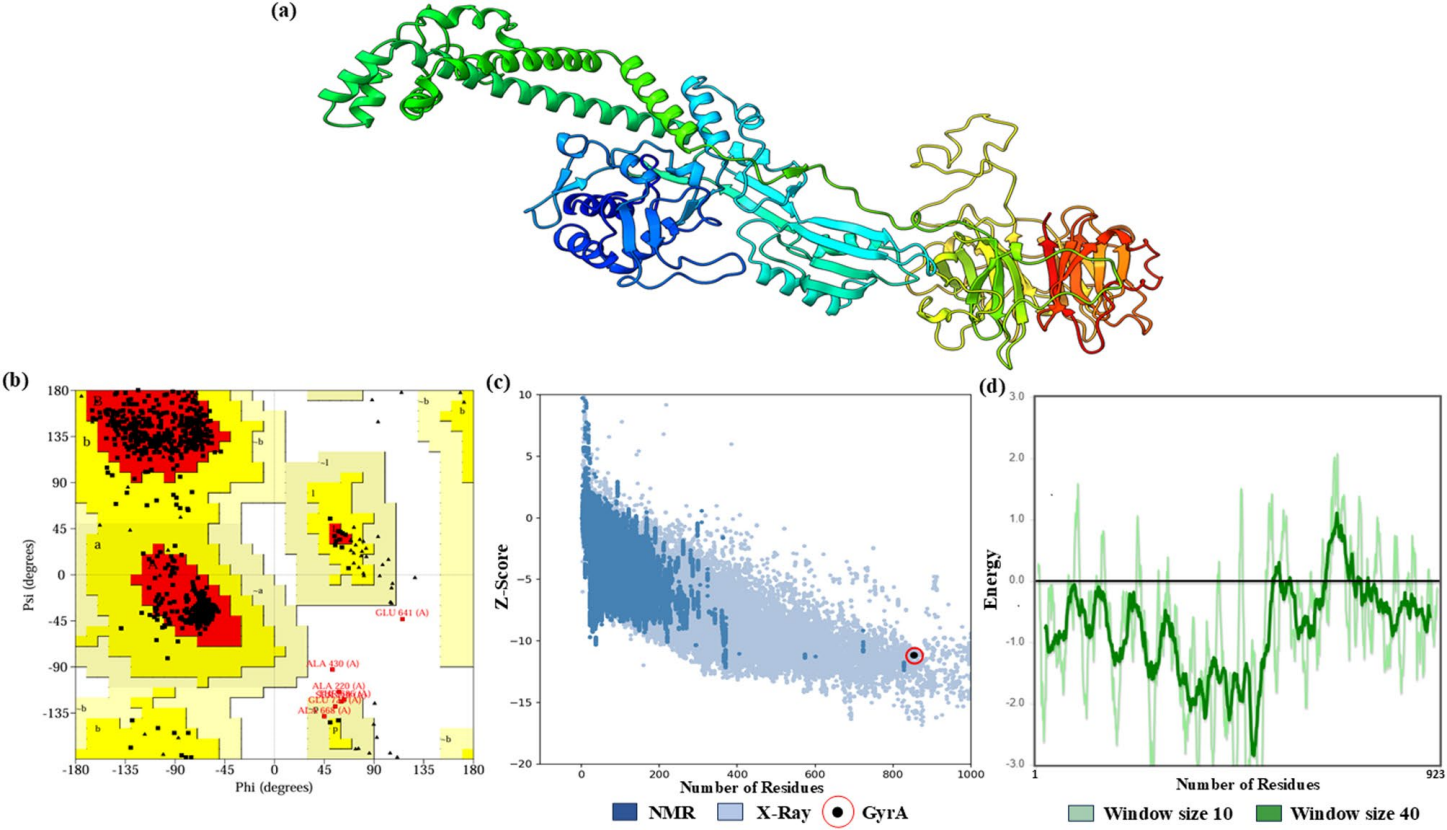
The structural analysis of modeled protein gyrA. (**a**) Optimized modeled 3D structure of gyrA. (**b**) Ramachandran plot of modeled protein. (**c**) Global model quality. (**d**) Local model quality.

The refined 3D modeled structure of gyrA (Fig. 1a) was optimized and validated using PROCHECK, ERRAT, and the ProSA-Web tool (an interactive web-based service for protein structure analysis). Final refinement of the modeled structure revealed 93.4% and 5.7% of residues in the most favorable regions and additionally allowed regions, respectively, with 0.4% of residues residing in the disallowed region in the Ramachandran plot and a 95.45% average overall quality factor (by ERRAT), indicating the conformational accuracy and quality of the generated modeled protein structure. Figure 1b. The theoretical framework suggests that structures with superior resolution typically exhibit values at or exceeding 95%^26^. The global model quality assessment of the predicted protein’s gross structural characteristics yielded a Z-score of – 11.16. This value aligns closely with the range typically observed in protein structures experimentally determined by X-ray crystallography (Fig. 1c). The local model quality, expressed in terms of stable protein conformation, was validated based on the energetics curve and topoisomerase (TOPO) IIA-type catalytic domain of gyrA, which was below the stability threshold cutoff (0.00) (Fig. 1d).

### Structural stability analysis of target protein and selected mutants

**Fig. 2:**
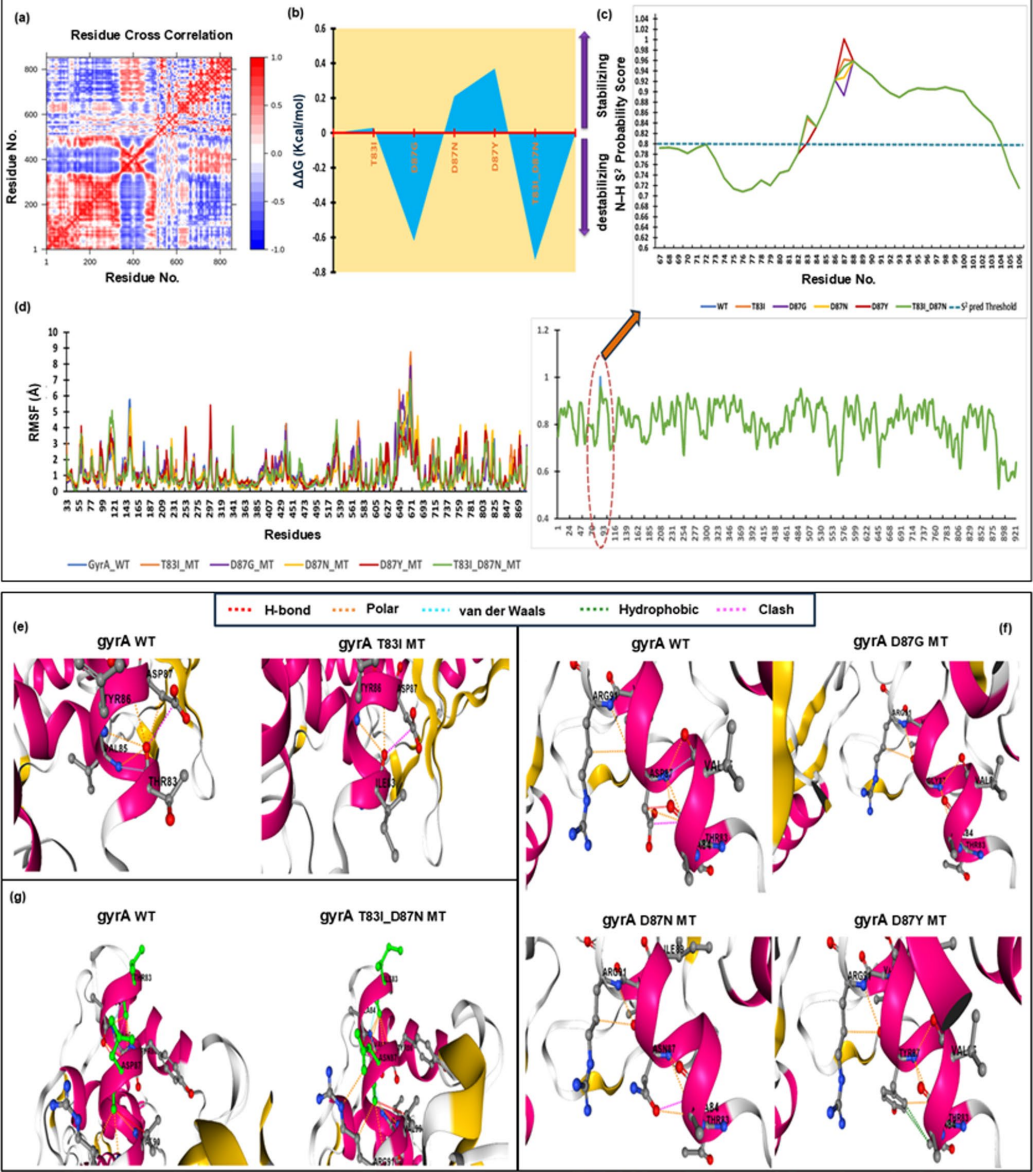
Structural stability and flexibility analysis of gyrA wild (WT) and Mutants (MTs). (**a**) DCCM plot of gyrA WT. (**b**) Stabilizing and destabilizing mutations of all the mutants in the QRDR domain of gyrA protein. (**c**) Backbone flexibility in gyrA WT and all MTs, major focusing on QRDR domain (67–106). (**d**) The relative residue level fluctuation profile of gyrA WT and all MTs. Loss, and gain of intra-chain interactions due to mutations (**e**) gyrA WT and gyrA T83I MT (**f**) gyrA WT, gyrA D87G MT, gyrA D87N MT and gyrA D87Y MT (**g**) gyrA WT and gyrA T83I_D87N MT.

In NMA, based on the DCCM plot (Fig. 2a), the red regions indicate strong positive correlations between the residue fluctuations. This observation suggests that these residues exhibit coordinated movement, potentially forming stable structural elements within the protein. Conversely, the blue regions demonstrate strong negative correlations, implying that these residues move in opposite directions, which may indicate flexible regions or hinges within the protein structure^27^. The prominent diagonal line represents the self-correlation of each residue with itself, which was expected to be high. This suggests a complex interplay between residues, with both cooperative and opposing motions contributing to protein function. The mutation T83I, D87N and D87Y showed a stabilizing effect (∆∆G = 0.03, 0.21, and 0.37 kcal/mol repectively) whereas mutation D87G (∆∆G = – 0.62 kcal/mol) and double mutation T83I_D87N (∆∆G = – 0.73 kcal/mol) conferred destability (Table 1) (Fig. 2b).

**Table 1:** Predicted stability changes due to mutations.

gyrA mutants	ΔΔG (Predicted stability change) kcal/mol	Stabilizing/destabilizing
T83I	0.03	Stabilizing
D87G	– 0.62	Destabilizing
D87N	0.21	Stabilizing
D87Y	0.37	Stabilizing
T83I_D87N	– 0.73	Destabilizing

### Protein backbone dynamics and folding free energy determination analysis

The relative backbone dynamics of the gyrA wild type (gyrA_WT) and its mutants (MTs) demonstrated no significant overall alteration in the average S^2^-probability score (∼0.807). The S^2^-probability scores of the whole protein and topoisomerase (TOPO) IIA-type catalytic domain (residues 34–534) were > 0.8, validating the structural integrity of the protein. However, when the specific mutations were considered, T83I of gyrA increased the S^2^-probability score from 0.802 to 0.848, in D87G, D87N and D87Y changed from 0.962 to 0.893, 0.927 and 1.001 respectively whereas in double mutants T83I_D87N, T83I increased from 0.802 to 0.854 and D87N reduced from 0.962 to 0.948 (Supplementary File 1). The overall rigidity of the concerned topoisomerase (TOPO) IIA-type catalytic domain and in between QRDR remained almost the same (changes less than 0.1%) (Fig. 2c). The average RMSFs of gyrA_WT and all the mutants were depicted in Fig. 2d. The localized enhancement in molecular flexibility resulting from the reduction of intra-chain interactions, which is a consequence of the decreased accessible surface area, contrasts with the diminished flexibility of side chains observed when intra-chain interactions increase. Mutations can induce comprehensive alterations in protein dynamics through the acquisition and loss of intra-chain interactions, increasing and reducing accessible surface area (Fig. 2e–g).

### Ligand-based analog screening, pharmacokinetics and antibacterial property prediction

Pharmacokinetic evaluation of individual compounds was conducted using the SwissADME web interface. A detailed compilation of the ADME properties of the 264 lead-like analogs is available in Supplementary File 2.1. Among the 264 compounds analyzed, 146 demonstrated compliance with the key pharmacokinetic criteria, including Lipinski’s rule. These characteristics indicated favorable absorption, efficient renal clearance, and synthetic feasibility, suggesting the potential therapeutic utility of the identified phytochemicals (Supplementary file 2.2). The screening process employed drug-likeness criteria and additional medicinal chemistry parameters to narrow down the selection of compounds most appropriate for therapeutic interventions. Additionally, toxicity assessment conducted using the ProtoxIII server yielded parameters indicating that the selected compounds exhibited relatively low toxicity [Classes 4–5 (with Class 1 representing the highest toxicity and Class 6 the lowest)]^28^. A total of 42 compounds met the established toxicity parameters. The selected compounds exhibited no evidence of carcinogenicity or mutagenicity. The toxicity assessment encompassed hepatotoxicity, nephrotoxicity, cardiotoxicity, carcinogenicity, mutagenicity, cytotoxicity, and additional cellular toxicity parameters (Supplementary File 2.3). A set of five mutant types (MTs) of gyrA, along with its wild-type, were individually docked with 42 compounds that met the ADMET property criteria. The binding affinities of the analogs and gyrA variants are shown in Table 2. Subsequently, three compounds exhibiting the highest average binding energies were identified for a more comprehensive evaluation. Table 3 provides a detailed comparison of the control antibiotics ciprofloxacin and levofloxacin, along with the three most promising lead compounds (M2, M9 and M11). In silico predictions revealed that all three compounds exhibited promising antibacterial activity along with various physicochemical attributes, pharmacokinetic properties, drug-likeness characteristics, and other biological effects (Table 4). The results of the toxicity evaluation of the control antibiotics and the three most promising lead compounds are presented in Table 5.

**Table 2:** Result from virtual screening.

Ligands	gyrA (WT)	D87G (MT)	D87N (MT)	D87Y (MT)	T83I (MT)	D87N_T83I (MT)	Average BE (kcal/mol)
CIP	– 6.6	– 6.3	– 6.1	– 6.2	– 6.1	– 6.1	– 6.23
LEV	– 7.4	– 5.8	– 6	– 6	– 6	– 5.8	– 6.17
M1	– 4.7	– 5.4	– 5.4	– 4.9	– 5.6	– 4.7	– 5.11
**M2**	**– 7.4**	**– 7**	**– 7.5**	**– 6.4**	**– 7.5**	**– 7.5**	**– 7.22**
M3	– 5.5	– 7.2	– 5.5	– 6.4	– 6.4	– 5.5	– 6.08
M4	– 6.3	– 6.1	– 6.1	– 5.3	– 5.4	– 6.1	– 5.88
M5	– 6.4	– 6.2	– 6.2	– 6.4	– 6.4	– 6.2	– 6.30
M6	– 5.6	– 5.2	– 5.6	– 5.9	– 5.5	– 5.6	– 5.57
M7	– 5.3	– 5.2	– 5.2	– 5.5	– 5.1	– 5.2	– 5.25
M8	– 5.6	– 5.4	– 5.6	– 5.7	– 5.6	– 5.5	– 5.57
**M9**	**– 6.5**	**– 6.7**	**– 6.3**	**– 6.6**	**– 6.4**	**– 6.5**	**– 6.50**
M10	– 6.1	– 6.3	– 6.1	– 6.2	– 6.3	– 6.1	– 6.18
**M11**	**– 7**	**– 7.3**	**– 7.1**	**– 6.6**	**– 6.5**	**– 7**	**– 6.92**
M12	– 5.6	– 5.7	– 5.6	– 5.6	– 5.6	– 5.6	– 5.62
M13	– 5.4	– 5.5	– 5.6	– 5.9	– 5.4	– 5.6	– 5.57
M14	– 5.8	– 5.6	– 5.5	– 5.8	– 5.4	– 5.6	– 5.62
M15	– 5.5	– 5.7	– 5.8	– 5.7	– 5.6	– 5.4	– 5.62
M16	– 5.9	– 5.7	– 5.9	– 6.2	– 5.8	– 5.9	– 5.90
M17	– 5.8	– 5.7	– 5.2	– 6.1	– 5.8	– 5.2	– 5.63
M18	– 5.5	– 5.4	– 5.4	– 5.6	– 5.5	– 5.4	– 5.47
M19	– 5.7	– 5.7	– 5.7	– 6	– 5.7	– 5.8	– 5.77
M20	– 5.8	– 5.4	– 5.9	– 6.2	– 5.8	– 5.9	– 5.83
M21	– 5.2	– 5.1	– 5.2	– 5.5	– 5.1	– 5.2	– 5.22
M22	– 5.4	– 5.5	– 5.4	– 5.7	– 5.6	– 5.4	– 5.50
M23	– 6	– 5.9	– 6	– 6	– 6	– 6	– 5.98
M24	– 5.4	– 6	– 6.1	– 5.4	– 6.1	– 5.2	– 5.70
M25	– 6	– 5.9	– 6	– 6.4	– 6.1	– 6	– 6.07
M26	– 4.9	– 5	– 5	– 5.2	– 4.7	– 5	– 4.97
M27	– 5.4	– 5.4	– 5.4	– 5.9	– 5.4	– 5.4	– 5.48
M28	– 5.7	– 5.6	– 5.7	– 5.8	– 5.7	– 5.7	– 5.70
M29	– 5.4	– 5.5	– 5.3	– 5.6	– 5.3	– 5.3	– 5.40
M30	– 5.3	– 5.3	– 5.1	– 5.5	– 5.2	– 5.3	– 5.28
M31	– 5.4	– 5.1	– 5.5	– 5.6	– 5.4	– 5.2	– 5.36
M32	– 5.2	– 5.4	– 5.4	– 5.5	– 5.4	– 5.4	– 5.38
M33	– 5.4	– 5.4	– 5.4	– 5.6	– 5.6	– 5.7	– 5.52
M34	– 5.5	– 5.5	– 5.5	– 5.4	– 5.5	– 5.5	– 5.48
M35	– 5.8	– 5.8	– 5.7	– 5.7	– 5.8	– 5.7	– 5.75
M36	– 5.7	– 5.7	– 5.6	– 5.9	– 5.7	– 5.7	– 5.72
M37	– 6.7	– 6.1	– 6.5	– 6.4	– 6.6	– 6.5	– 6.47
M38	– 4.7	– 5.2	– 4.8	– 4.8	– 4.6	– 4.7	– 4.80
M39	– 5.2	– 5.1	– 5.1	– 5.5	– 5.2	– 5.2	– 5.22
M40	– 5.1	– 5.2	– 5.1	– 5.3	– 5.1	– 5.1	– 5.15
M41	– 5.3	– 5	– 5	– 5.6	– 5.3	– 5.1	– 5.22
M42	– 4.8	– 5.1	– 5.1	– 5.1	– 5.1	– 5	– 5.03

**Table 3:** Details of control antibiotics and best 3 lead compounds.

Compound code	Name	PubChem ID/ZINC ID	Molecular Formula	Structure
CIP	Ciprofloxacin	2764/ZINC20220	C_17_H_18_FN_3_O_3_	
LEV	Levofloxacin	149096/ZINC538273	C_18_H_20_FN_3_O_4_	
M2	N-benzylquinoline-8-sulfonamide	3776856/ZINC000002779748	C_16_H_14_N_2_O_2_S	
M9	8-(Benzenesulfonylmethyl) quinoline	25640151/ZINC000012974785	C_16_H_13_NO_2_S	
M11	Quinolin-8-ylmethyl pyridine-2-carboxylate	41914274/ZINC000011297510	C_16_H_12_N_2_O_2_	

**Table 4:** Physiochemical properties, Pharmacokinetics, drug-likeness, medicinal chemistry and predicted antibacterial activity of control antibiotics and best 3 lead compounds.

Control Drugs/ Molecule	MW (g/mol)	TPSA (Å²)	Rotatable bonds	MLOGP	GI absorption	BBB permeant	Drug-property violations	Lead-likeliness violations	Synthetic accessibility	Predicted antibacterial property
CIP	331.34	74.57	3	1.28	High	No	No	No	2.51	Proven
LEV	361.37	75.01	2	0.98	High	No	No	Yes	3.63	Proven
M2	298.36	67.44	4	1.8	High	Yes	No	No	2.38	Yes
M9	283.34	55.41	3	2.65	High	Yes	No	Yes	2.73	Yes
M11	264.28	52.08	4	1.87	High	Yes	No	No	2.04	Yes

**Table 5:** Toxicity evaluation of control antibiotics and best 3 lead compounds.

Compound code	Predicted LD50 (mg/kg)	Predicted toxicity class	Hepatotoxicity	Nephrotoxicity	Cardiotoxicity	Carcinogenicity	Mutagenicity	Cytotoxicity
CIP	2000	4	Inactive	Active	Inactive	Inactive	Active	Inactive
LEV	1478	4	Inactive	Active	Inactive	Inactive	Active	Inactive
M2	2800	5	Inactive	Inactive	Inactive	Inactive	Inactive	Inactive
M9	1000	4	Inactive	Inactive	Inactive	Inactive	Inactive	Inactive
M11	1600	4	Inactive	Inactive	Inactive	Inactive	Inactive	Inactive

### Ligand optimization upon DFT simulation

**Fig. 3:**
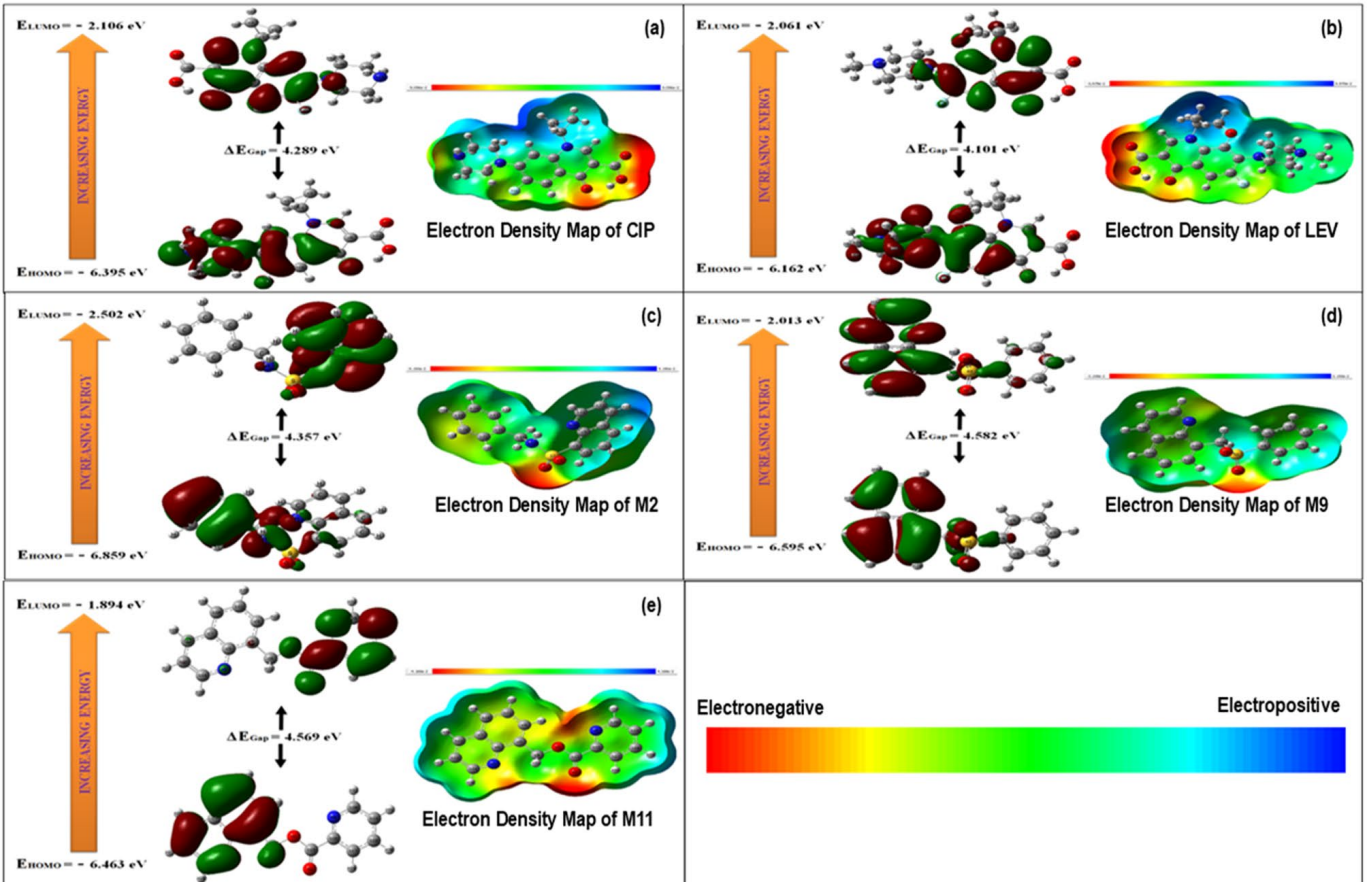
Frontier molecular orbital (HOMO-LUMO) and molecular electrostatic potential diagram of control and lead compounds from DFT simulation analysis using GaussView v6.1.1^66^. (**a**) Ciprofloxacin (CIP), (**b**) Levofloxacin (LEV), (**c**) M2, (**d**) M9, (**e**) M11.

Density Functional Theory (DFT) analysis investigated molecule properties, including electronic structure, chemical reactivity, and protein-drug molecule interactions, for the three most promising lead compounds^29^. The Frontier Molecular Orbital (FMO) theory was utilized to elucidate the electron donating (HOMO) and accepting (LUMO) capacities of these molecules. The energy difference between the LUMO and HOMO, known as the gap energy, offers insights into the stabilization interactions within protein-ligand complexes. A smaller HOMO-LUMO energy gap correlates with increased reactivity and decreased stabilization interactions between the ligand and the protein^30^. Among the compounds, M2 displayed a comparatively lower gap energy (4.357 eV), indicating high reactivity, whereas M9 (4.582 eV) and M11 (4.569 eV) exhibited higher (ΔE) gap energies than M2, suggesting greater stability. The M2 compounds also demonstrated minimum chemical potential, supporting their efficacy in protein interactions. The top three hits exhibited reduced chemical potential compared to the positive control, signifying enhanced stability and formation of stable receptor complexes. Molecular electrostatic potentials (MEPs) were employed to represent the relative polarity of the compounds visually by correlating their electronegativity, dipole moment, and molecular charge distribution (Fig. 3a–e). Notably, compound M2 exhibited higher electrophilicity, as evidenced by the more pronounced blue region in the figure. Table 6 shows the global reactivity parameters for the control antibiotics and the three most promising compounds identified in this study.

**Table 6:** Global reactivity parameters descriptions of control antibiotics and three best compounds in eV.

Compound code	HOMO	LUMO	ΔE	Ionization potential (I)	Electron affinity (A)	Electro-negativity (χ)	Chemical potential (µ)	Global hardness (η)	Softness (Ѕ)	Electrophilicity (ω)
Ciprofloxacin	– 6.395	– 2.106	4.289	6.395	2.106	4.250	– 4.250	2.144	0.466	4.211
Levofloxacin	– 6.162	– 2.061	4.101	6.162	2.061	4.111	– 4.111	2.050	0.487	4.121
M2	– 6.859	– 2.502	4.357	6.859	2.502	4.680	– 4.680	2.178	0.459	5.020
M9	– 6.595	– 2.013	4.582	6.595	2.013	4.304	– 4.304	2.291	0.436	4.042
M11	– 6.463	– 1.894	4.569	6.463	1.894	4.178	– 4.178	2.284	0.437	3.820

### Binding profiles of docked complexes

A molecular docking study was undertaken to elucidate potential inhibitors of gyrA proteins, encompassing both the WT and various MT forms. The study focused on single mutations T83I, D87G, D87N, and D87Y, as well as the double mutation T83I_D87N. The binding interactions of the target protein and its mutations with the quinoline analogs M2, M9, and M11 were investigated. Compounds that demonstrated binding to different mutation points in the quinolone resistance-determining region (QRDR) of gyrA were considered preferential in the docking analysis.

**Fig. 4:**
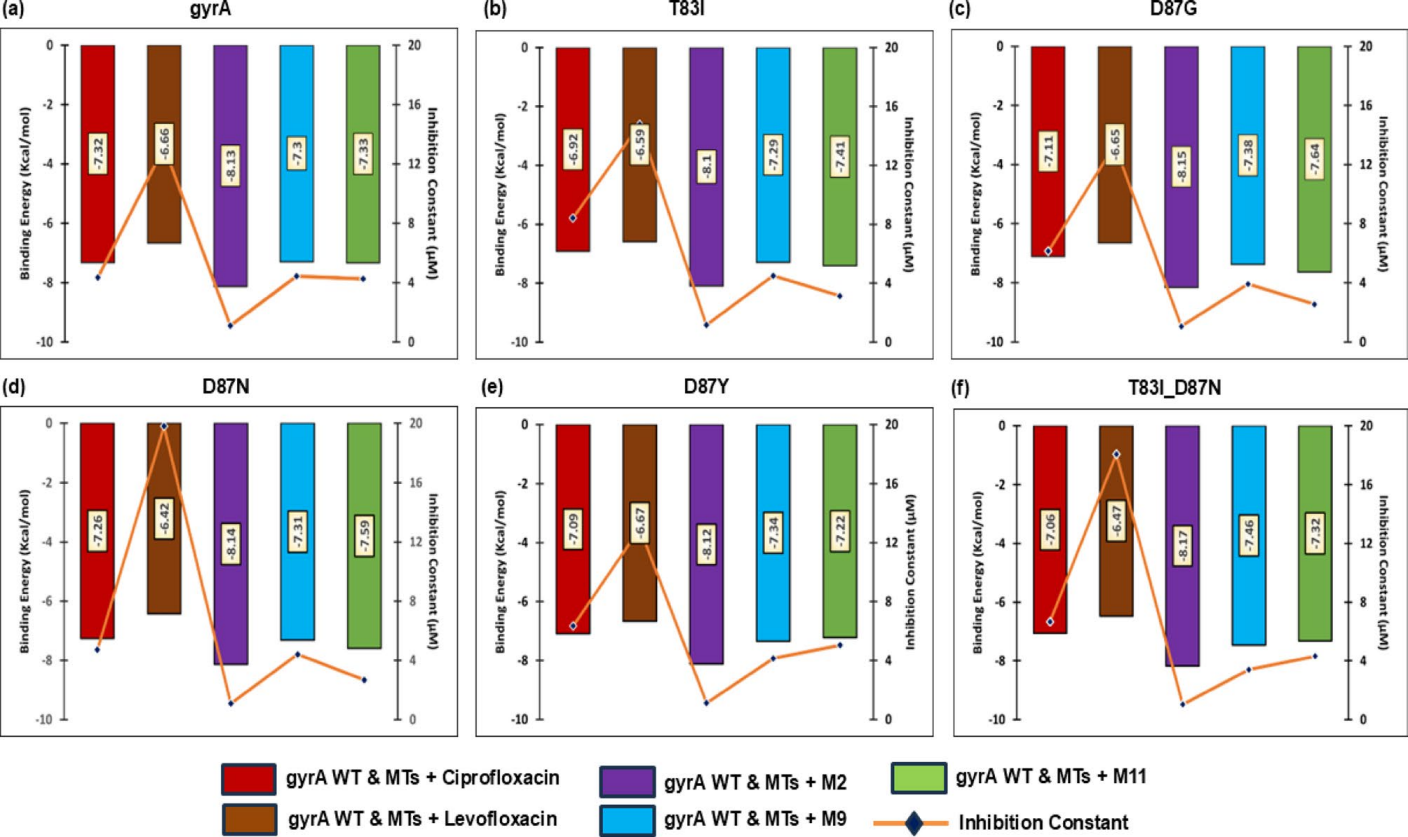
Molecular docking results- Binding energies and inhibition constants of all docked complexes of proteins (gyrA WT and all MTs) and ligands (CIP, LEV, M2, M9 and M11). (**a**) gyrA WT, (**b**) T83I, (**c**) D87G, (**d**) D87N, (**e**) D87Y, (**f**) T83I_D87N.

M2 exhibited an average binding affinity of − 8.14 kcal/mol, surpassing that of both the control antibiotics ciprofloxacin and levofloxacin. Following M2, M11 demonstrated the highest average binding affinity with WT & MTs of gyrA (-7.42 kcal/mol), followed by M9 (-7.35 kcal/mol), control ciprofloxacin (-7.13 kcal/mol), and levofloxacin (-6.58 kcal/mol), respectively. Figure 4a-f depicts the binding energy and inhibition constant patterns of control antibiotics and the three compounds M2, M9, and M11 against the gyrA wild-type and mutant proteins.

**Fig. 5:**
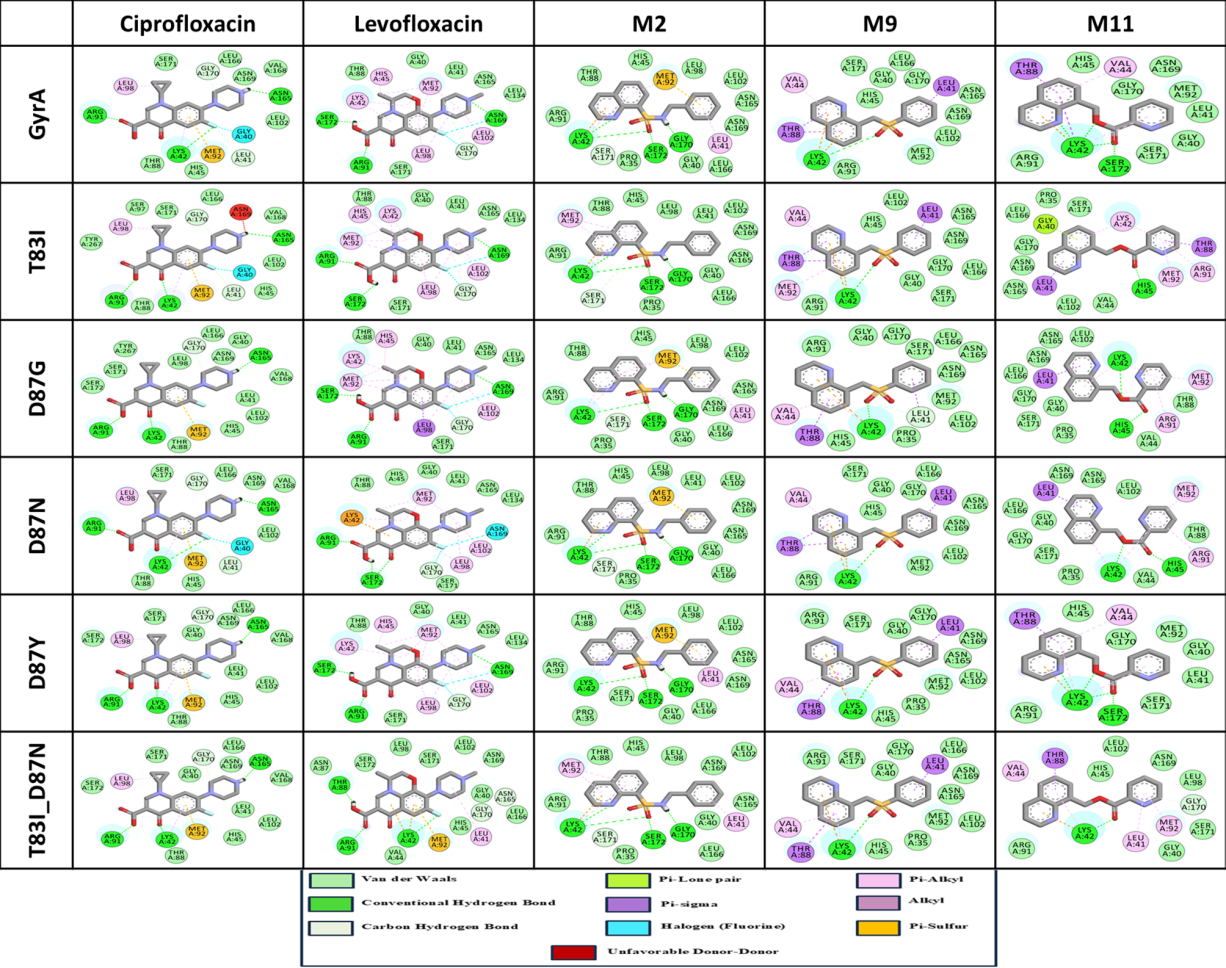
Intermolecular Interaction profiles of the gyrA WT and all MTs with CIP, LEV, M2, M9 and M11.

Table 7 presents a comprehensive analysis of the individual binding energies and inhibition constants of all complexes. The overall binding energies, with negligible standard deviation (SD), and inhibition constants of all complexes were provided in triplicate in Supplementary File 3. These findings demonstrate M2’s superior capacity to overcome resistance induced by mutations. Variations in drug-binding energies arise from alterations in intermolecular interactions with the WT and MT of gyrA proteins (Fig. 5).

**Table 7:** Relative binding energies (BE), inhibition constants, and interactions of GyrA WT and MTs with control antibiotics and best 3 lead compounds.

Ligands	Target proteins (WT & MTs)	Binding energy (kcal/mol)	Inhibition constant (µM)	Conventional H-bond	Van der Waals interactions
CIP	gyrA	– 7.32	4.35	Lys42, Arg91, Asn165	His45, Thr88, Leu102, Leu166, Val168, Asn169, Ser171
	gyrA_T83I	– 6.92	8.43	Lys42, Arg91, Asn165	His45, Thr88, Ser97, Leu102, Leu166, Val168, Ser171, Tyr267
	gyrA_D87G	– 7.11	6.14	Lys42, Arg91, Asn165	Gly40, Leu41, His45, Thr88, Leu98, Leu102, Leu166,Val168, Asn169, Ser171, ser172, Tyr267
	gyrA_D87N	– 7.26	4.73	Lys42, Arg91, Asn165	His45, Thr88, Leu102, Leu166,Val168, Asn169, Ser171, Ser172
	gyrA_D87Y	– 7.09	6.34	Lys42, Arg91, Asn165	Gly40, Leu41, His45, Thr88, Leu102, Leu166,Val168, Asn169, Ser171, Ser172, Tyr267
	gyrA_T83I_D87N	– 7.06	6.67	Lys42, Arg91, Asn165	Gly40, Leu41, His45, Thr88, Leu102, Leu166,Val168, Asn169, Ser171, Ser172
Average BE & Inh. const. of CIP		**– 7.13**	**6.11**		
LEV	gyrA	– 6.66	13.13	Arg91, Asn169, Ser172	Gly40, Leu41, Thr88, Leu134, Asn165, Ser171
	gyrA_T83I	– 6.59	14.76	Arg91, Asn169, Ser172	Gly40, Leu41, Thr88, Leu134, Asn165, Ser171
	gyrA_D87G	– 6.65	13.32	Arg91, Asn169, Ser172	Gly40, Leu41, Thr88, Leu134, Asn165, Ser171
	gyrA_D87N	– 6.42	19.8	Arg91, Asn169, Ser172	Gly40, Leu41, His45, Thr88, Leu134, Asn165, Ser171
	gyrA_D87Y	– 6.67	12.96	Arg91, Asn169, Ser172	Gly40, Leu41, Thr88, Leu134, Asn165, Ser171
	gyrA_T83I_D87N	– 6.47	18.08	Lys42, Thr88, Arg91	Gly40, Val44, His45, Asn87, Leu98, Leu102, Leu166, Asn169, Ser171, Ser172
Average BE & Inh. const. of LEV		**– 6.58**	**15.34**		
M2	gyrA	– 8.13	1.1	Lys42, Gly170, Ser172	Pro35, Gly40, His45, Thr88, Arg91, Leu98, Leu102, Asn165, Leu166, Asn169
	gyrA_T83I	– 8.1	1.16	Lys42, Gly170, Ser172	Pro35, Gly40, His45, Thr88, Arg91, Leu98, Leu102, Asn165, Leu166, Asn169
	gyrA_D87G	– 8.15	1.06	Lys42, Gly170, Ser172	Pro35, Gly40, His45, Thr88, Arg91, Leu98, Leu102, Asn165, Leu166, Asn169
	gyrA_D87N	– 8.14	1.09	Lys42, Gly170, Ser172	Pro35, Gly40, His45, Thr88, Arg91, Leu98, Leu102, Asn165, Leu166, Asn169
	gyrA_D87Y	– 8.12	1.12	Lys42, Gly170, Ser172	Pro35, Gly40, His45, Thr88, Arg91, Leu98, Leu102, Asn165, Leu166, Asn169
	gyrA_T83I_D87N	– 8.17	1.03	Lys42, Gly170, Ser172	Pro35, Gly40, His45, Thr88, Arg91, Leu98, Leu102, Asn165, Leu166, Asn169
Average BE & Inh. const. of M2		**– 8.14**	**1.09**		
M9	gyrA	– 7.3	4.44	Lys42	Gly40, His45, Arg91, Met92, Asn165, Leu166, Asn169, Gly170, Ser171
	gyrA_T83I	– 7.29	4.51	Lys42	Gly40, His45, Arg91, Leu102, Asn165, Leu166, Asn169, Gly170, Ser171
	gyrA_D87G	– 7.38	3.92	Lys42	Pro35, Gly40, His45, Arg91, Met92, Leu102, Asn165, Leu166, Asn169, Gly170, Ser171
	gyrA_D87N	– 7.31	4.41	Lys42	Gly40, His45, Arg91, Met92, Leu102, Asn165, Leu166, Asn169, Gly170, Ser171
	gyrA_D87Y	– 7.34	4.15	Lys42	Pro35, Gly40, His45, Arg91, Met92, Leu102, Asn165, Asn169, Gly170, Ser171
	gyrA_T83I_D87N	– 7.46	3.41	Lys42	Pro35, Gly40, His45, Arg91, Met92, Leu102, Asn165, Leu166, Asn169, Gly170, Ser171
Average BE & Inh. const. of M9		**– 7.35**	**4.14**		
M11	gyrA	– 7.33	4.26	Lys42, Ser172	Gly40, Leu41, His45, Arg91, Met92, Asn169, Gly170, Ser171
	gyrA_T83I	– 7.41	3.13	His 45	Pro35, Val44, Leu102, Asn165, Leu166, Asn169, Gly170, Ser171
	gyrA_D87G	– 7.64	2.54	Lys42, His45	Pro35, Gly40, Val44, Thr88, Leu102, Asn165, Leu166, Asn169, Gly170, Ser171
	gyrA_D87N	– 7.59	2.68	Lys42,His45	Pro35, Gly40, Val44, Thr88, Leu102, Asn165, Leu166, Asn169, Gly170, Ser171
	gyrA_D87Y	– 7.22	5.03	Lys42, Ser172	Gly40, Leu41, His45, Arg91, Met92, Gly170, Ser171
	gyrA_T83I_D87N	– 7.32	4.32	Lys42	Gly40, His45, Arg91, Leu98, Leu102, Asn169, Ser171
Average BE & Inh. const. of M11		**– 7.42**	**3.6**		

Compound M2 demonstrated superior inhibitory properties, exhibiting the lowest average inhibition constant (1.09 µM) against wild-type and mutant gyrA. In comparison, M9 and M11 displayed average inhibition constants of 4.14 µM and 3.6 µM, respectively, which were notably lower than those of the control antibiotics ciprofloxacin (13.32 µM) and levofloxacin (15.34 µM). All three compounds (M2, M9, and M11) exhibited higher binding energies and lower inhibition constants than ciprofloxacin and levofloxacin. The data apparently indicated that M2 was the most efficacious compound among the tested substances, including the control antibiotics. Moreover, the exceptionally favorable inhibition constants observed for M2 (with an average of 1.09 µM) corroborated its status as a potent gyrA inhibitor. These findings underscore the necessity for further investigation using md simulations to assess the dynamic behavior and stability of M2.

### Molecular dynamics simulation for structural stability assessment

**Fig. 6:**
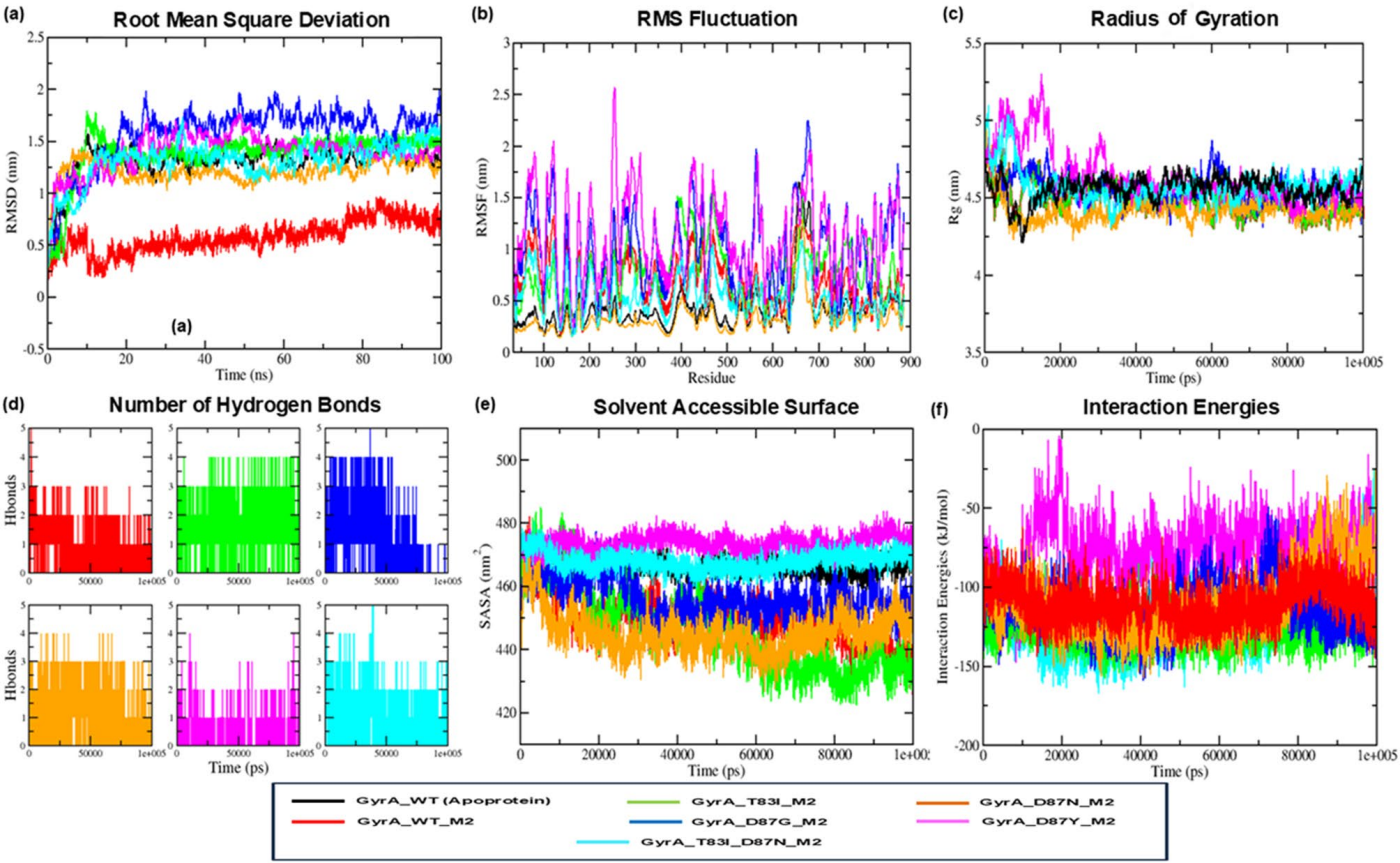
Structural stability assessment of apoprotein gyrA wild and all complexes of gyrA WT and MTS with M2 through MDS. (**a**) RMSD profile, (**b**) Residue level RMSF plot. (**c**) Radius of gyration. (**d**) No. of hydrogen bonds. (**e**) Solvent accessible surface area profile. (**f**) Interaction energy profile.

The stability and dynamic interactions of the M2-gyrA WT and MTs complexes were analyzed using molecular dynamics simulations. These findings revealed that M2 established highly stable complexes with both WT and MTs gyrA proteins when subjected to physiological conditions. The M2-bound complexes exhibited consistent RMSD values with minimal variations, indicating stable and robust interactions. The analysis of M2 binding to gyrA_WT, T83I_MT, D87G_MT, D87N_MT, D87Y_MT, and T83I_D87N_MT revealed average RMSD values of 0.576, 1.379, 1.571, 1.210, 1.392, and 1.318 nm, respectively. In comparison, the apoprotein gyrA had an RMSD of 1.305 nm. However, the gyrA_WT complex with M2 exhibited a comparatively low average RMSD of 0.576 nm, showing reduced fluctuations compared to the mutated protein complexes (Fig. 6a). Analysis of the MTs protein complexes revealed overall backbone fluctuations extending up to (∼0.7 nm). The D87G_MT and D87Y_MT variants demonstrated comparatively higher fluctuations than the apoprotein gyrA, gyrA_WT_M2, and other MTs_M2 complexes, as illustrated in (Fig. 6b).

The radius of gyration (Rg) exhibited minimal changes (∼0.1 nm) in all complexes compared to apoprotein gyrA, indicating similar compactness even after binding with compound M2. In the D87Y_MT_M2 complex, Rg exhibited a minimal increase (∼ 0.1 nm) relative to the apoprotein gyrA (Fig. 6c).

Throughout the simulation, the consistent hydrogen bonding observed across all docked complexes played a crucial role in determining the binding affinity between M2 and gyrA proteins (WT and MTs). Analysis revealed that gyrA_WT maintained two out of five total hydrogen bonds, while the M2 complexes with T83I_MT, D87G_MT, D87N_MT, D87Y_MT, and T83I_D87N_MT exhibited four out of four, one out of five, three out of four, two out of four, and three out of five consistent hydrogen bonds, respectively (Fig. 6d). These findings demonstrate that M2 sustained a consistent number of interactions with critical residues thus imparting its stable binding profile with the target protein throughout the studied timeframe of 100 ns.

Compactness analyses as understood from Rg profile was further validated from the SASA trajectories which revealed the formation of uniform layer of solvation to the studies complexes. The complexes gyrA_WT_M2 (449.43 nm^2^, T83I_MT_M2 (446.25 nm^2^, D87G_MT_M2 (456.16 nm^2^, and D87N_MT_M2 (445.53 nm^2^ revealed a marginal decrease in the SASA area relative to the apoprotein (466.29 nm^2^. Conversely, D87Y_MT_M2 (473.69 nm^2^ and T83I_D87N_MT_M2 (467.95 nm^2^ exhibited a slight increase in SASA area (Fig. 6e).

Interaction energy (IE) profiles demonstrated favorable binding between the M2 compound and both WT and MT protein variants. The total IE for the gyrA_WT_M2 complexes was calculated to be – 110.893 kcal/mol . The MT complexes exhibited diverse IE values: T83I_MT_M2 at – 127.238 kcal/mol, D87G_MT_M2 at – 114.534 kcal/mol, D87N_MT_M2 at – 110.021 kcal/mol, D87Y_MT_M2 at – 76.675 kcal/mol, and T83I_D87N_MT_M2 at – 121.136 kcal/mol (Fig. 6f). The complete molecular dynamics simulation (MDS) results are provided in Supplementary File 4.

These findings demonstrate M2’s ability to effectively inhibit both WT and MT gyrA proteins, even in the presence of resistance-associated mutations.

### Validation of docked complexes energy profiles through MM/GBSA and MM/PBSA analysis

**Fig. 7:**
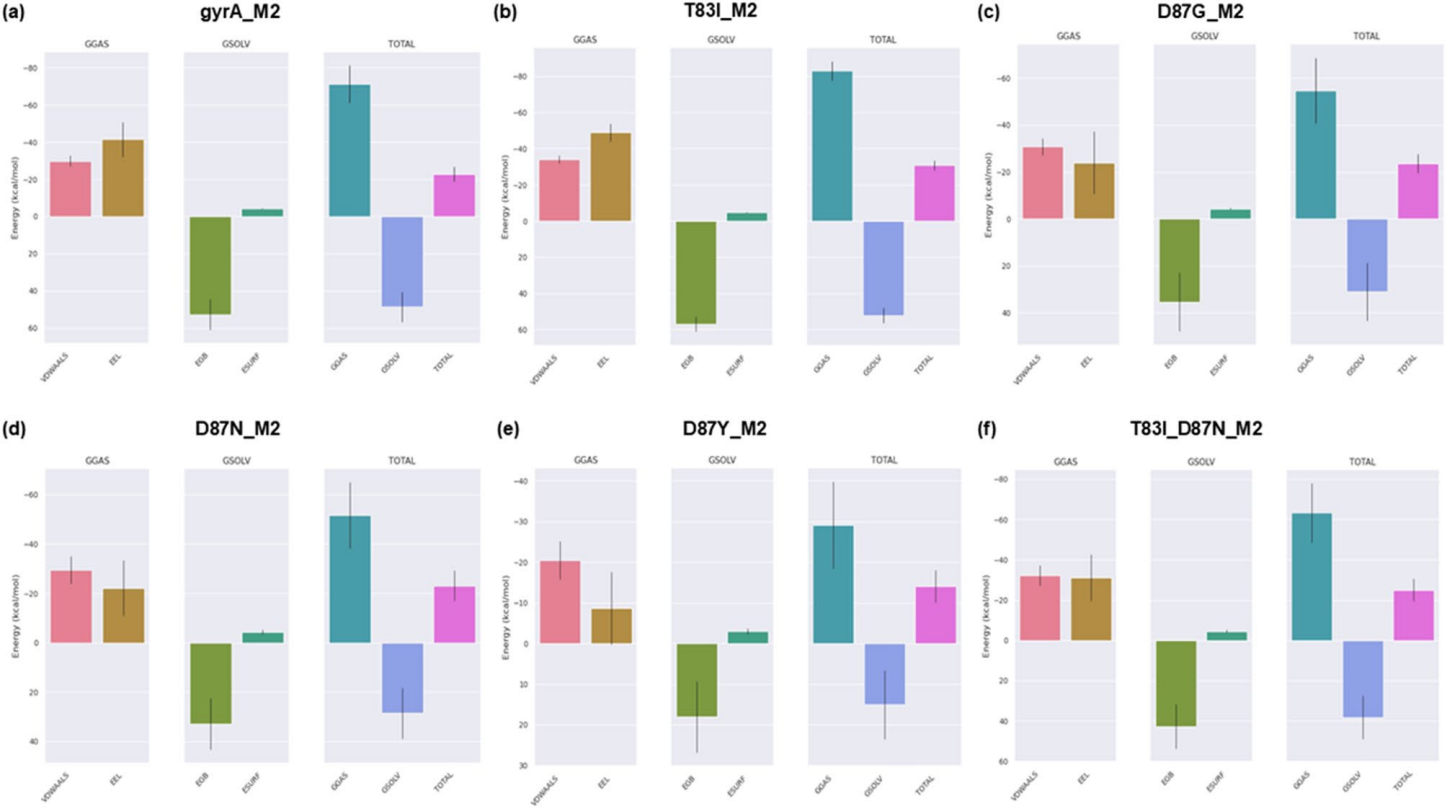
MMGBSA binding-free energy contribution of various interactions between the proteins gyrA WT and MTs with the lead compound M2. (**a**) gyrA_M2, (**b**) T83I_M2, (**c**) D87G_M2, (**d**) D87N_M2, (**e**) D87Y_M2, (**f**) T83I_D87N_M2.

The MM/GBSA and MM/PBSA method, employed in binding free energy calculations, offered a comprehensive thermodynamic analysis of the interactions between M2-gyrA WT and MTs. Upon achieving complex stabilization, the MM/GBSA and MM/PBSA binding energies were calculated for the protein-ligand complexes Tables 8a and 8b provide the binding free energy calculation of all the protein-ligand complexes using MM/GBSA and MM/PBSA methods respectively. From, the MM/GBSA and MM/PBSA analysis, yielded total binding energies (ΔG_bind) of − 22.63, – 30.82, – 23.55, – 23.11, – 14.02, – 25.06 kcal/mol and − 14.19, – 22.18, – 16.36, – 15.85, – 12.34, – 15.38 kcal/mol respectively for the complexes gyrA_WT, T83I_MT, D87G_MT, D87N_MT, D87Y_MT, and T83I_D87N_MT, respectively, when interacting with M2. Figure 7a–f presents binding-free energy contribution of various interactions between the proteins (WT and MTs) with the lead compound M2. This binding free energy calculated encompassing VDWAALS, EEL, EGB/EPB and ESURF/ENPOLAR. The MM/GBSA and MM/PBSA results corroborated the molecular docking and MD simulation findings, establishing M2 as a potent inhibitor capable of targeting resistant strains with high efficacy. The observed thermodynamic parameters elucidate the potential of M2 as a promising lead compound for future optimization and evolution into a therapeutic candidate against fluoroquinolone-resistant *P. aeruginosa* strains.

**Table 8a:** Calculations of protein-ligand binding energetics using the MM/GBSA method.

Complex	ΔVDWAALS	ΔEEL	ΔEGB	ΔESURF	ΔGGAS	ΔGSOLV	TOTAL (ΔG_bind)
gyrA_M2	– 29.88 ± 2.92	– 41.43 ± 9.38	52.9 ± 8.24	– 4.22 ± 0.31	– 71.31 ± 10.11	48.68 ± 8.1	– 22.63 ± 3.95
T83I_M2	– 34.07 ± 2.25	– 48.94 ± 4.89	57 ± 4.25	– 4.82 ± 0.2	– 83.01 ± 5.33	52.19 ± 4.18	– 30.82 ± 2.78
D87G_M2	– 30.7 ± 3.54	– 23.89 ± 13.29	35.41 ± 12.51	– 4.37 ± 0.46	– 54.6 ± 13.97	31.04 ± 12.37	– 23.55 ± 3.91
D87N_M2	– 29.53 ± 5.51	– 22.16 ± 11.16	32.87 ± 10.49	– 4.29 ± 0.84	– 51.68 ± 13.41	28.58 ± 10.26	– 23.11 ± 6.2
D87Y_M2	– 20.45 ± 4.74	– 8.68 ± 8.9	18.09 ± 8.8	– 2.98 ± 0.66	– 29.13 ± 10.68	15.11 ± 8.51	– 14.02 ± 3.98
T83I_D87N_M2	– 32.2 ± 5.11	– 31.1 ± 11.45	42.73 ± 11.12	– 4.48 ± 0.66	– 63.31 ± 14.8	38.25 ± 10.69	– 25.06 ± 5.41

**Table 8b:** Calculations of protein-ligand binding energetics using the MM/PBSA method.

Complex	ΔVDWAALS	ΔEEL	ΔEPB	ΔENPOLAR	ΔGGAS	ΔGSOLV	TOTAL (ΔG_bind)
gyrA_M2	– 29.95 ± 3.11	– 41.11 ± 9.26	60.12 ± 8.47	– 3.25 ± 0.15	– 71.06 ± 10.15	56.87 ± 8.40	– 14.19 ± 4.37
T83I_M2	– 34.07 ± 2.03	– 48.99 ± 4.74	64.05 ± 4.94	– 3.18 ± 0.10	– 83.05 ± 5.04	60.87 ± 4.91	– 22.18 ± 1.94
D87G_M2	– 30.83 ± 3.63	– 23.47 ± 12.84	41.22 ± 12.76	– 3.28 ± 0.19	– 54.30 ± 13.78	37.94 ± 12.72	– 16.36 ± 3.68
D87N_M2	– 29.53 ± 5.52	– 22.07 ± 11.24	38.98 ± 12.41	– 3.24 ± 0.39	– 51.60 ± 13.52	35.74 ± 12.23	– 15.85 ± 4.45
D87Y_M2	– 20.25 ± 4.62	– 8.82 ± 9.01	19.14 ± 9.98	– 2.41 ± 0.47	– 29.07 ± 10.95	16.73 ± 9.70	– 12.34 ± 3.20
T83I_D87N_M2	– 32.30 ± 4.85	– 30.72 ± 12.63	51.12 ± 15.09	– 3.48 ± 0.38	– 63.02 ± 15.76	47.64 ± 14.83	– 15.38 ± 5.38

### Principal component analysis

**Fig. 8:**
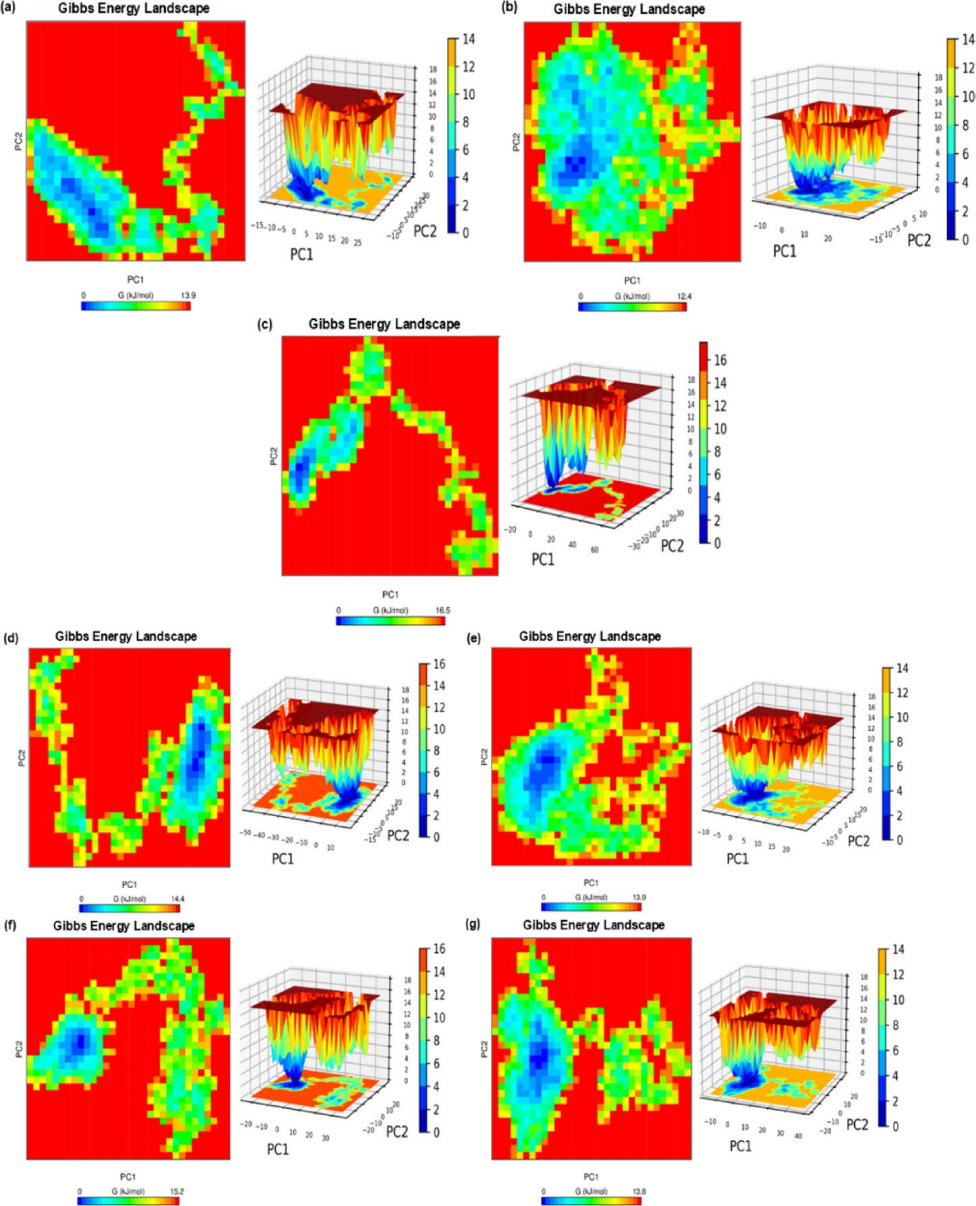
2D and 3D Gibbs free energy landscapes of GyrA apoprotein and all the complexes. (**a**) Apoprotein gyrA, (**b**) gyrA WT_M2, (**c**) T83I_M2, (**d**) D87G_M2, (**e**) D87N_M2, (**f**) D87Y_M2, (**g**) T83I_D87N_M2.

Principal Component Analysis (PCA) was utilized to assess the metastable conformational spaces of gyrA WT and MTs proteins in conjunction with the lead compound M2. The analysis of Gibbs free energy landscapes, specifically through the projection of the initial two principal components (PC1 and PC2), provided insights into the stability and dynamic interactions between proteins and ligands under physiologically relevant conditions. Figure 8 illustrates the 2D and 3D Gibbs free energy landscapes for each complex and apoprotein gyrA. The analysis identified global energy minima across all complexes, with distinct basins indicating stable protein conformation. Energetically favorable configurations are manifested as blue regions, whereas red regions denote higher-energy states. The apoprotein gyrA exhibited a broad energy landscape with multiple shallow basins, indicating high conformational flexibility in the absence of a ligand, which is characteristic of unbound proteins exploring a range of metastable states. Conversely, the gyrA_WT_M2 complex displayed a narrow, deep-energy well, suggesting a stable and energetically favorable conformation. This stabilization implies that M2 binding significantly reduces the conformational entropy, promoting a compact and stable protein structure. For the T83I mutant, the Gibbs free energy landscape was comparable to that of the gyrA_WT_M2 complex, with a deep energy well but relatively higher energy states, highlighting M2’s strong binding and stabilizing effect on the T83I mutant. The D87N mutant complex also exhibited a moderately deep energy well, indicating that M2 effectively stabilized this mutation with minimal impact on the overall protein stability. In contrast, the D87G and D87Y mutants exhibited broader basins and shallower energy minima, suggesting reduced conformational stability and greater structural variability. The double mutant T83I_D87N displayed an intermediate landscape, with an energy profile that was less stable than the T83I complex, but more stable than the single mutants D87G and D87Y. The PCA results demonstrated M2’s capacity to stabilize both WT and MTs gyrA proteins. These insights reinforce M2’s potential as a lead compound for combating fluoroquinolone-resistant *P. aeruginosa*.

## Discussion

Fluoroquinolone-resistant *P. aeruginosa* presents a significant public health concern, particularly in healthcare settings. This opportunistic pathogen has developed resistance against multiple classes of antibiotics, rendering infections challenging to treat^31^. One of the primary mechanisms of resistance in *P. aeruginosa* is the acquisition of mutations in the quinolone resistance-determining regions (QRDRs) of genes encoding DNA gyrase (gyrA) and topoisomerase IV (parC), which are the most extensively exploited and favorable targets of fluoroquinolones^12,32^. Fluoroquinolones are a class of antibiotics widely used to treat infections caused by *P. aeruginosa*. They target bacterial DNA gyrase and topoisomerase IV, which are essential for DNA replication and repair. Mutations in the QRDRs of these enzymes can alter their structure and function, thereby preventing fluoroquinolones from binding and inhibiting their activity. This leads to fluoroquinolone resistance. These mutations in the QRDRs can lead to various levels of resistance, ranging from low-level to high-level resistance^10^. In the present study, commonly observed and reported mutations in gyrA-T83I, D87G, D87N, D87Y, and the double mutation T83I_D87N for fluoroquinolone-resistant *P. aeruginosa* were identified^33–35^. Significant resistance to fluoroquinolone antibiotics (primarily ciprofloxacin and levofloxacin) was observed in these isolates. T83I and D87N have been previously reported to decrease the efficacy of the fluoroquinolone antibiotics ciprofloxacin and levofloxacin. These frequently reported mutations are located in the QRDR of the topoisomerase (TOPO) IIA-type catalytic domain of gyrA. This study assessed the quinoline analog M2 as a potential inhibitor of wild-type (WT) and mutant (MT) gyrA proteins, including T83I, D87G, D87N, D87Y, and T83I_D87N. Computational methodologies, including molecular docking, MD simulations, MM/PBSA free energy analysis, and PCA, were employed to assess the binding affinity, stability, and conformational dynamics of these complexes. Molecular docking analyses revealed that M2 demonstrated high binding affinity to both WT and MTs of gyrA, with a mean binding energy (BE) of − 8.14 kcal/mol, surpassing ciprofloxacin (avg. BE − 7.13) and levofloxacin (avg. BE − 6.58) in interactions with resistance-associated residues. M9 and M11 also displayed superior BE averages of – 7.35 and − 7.42 kcal/mol, respectively, compared to control antibiotics. M2 exhibited superior inhibitory properties, with the lowest average inhibition constant (1.09 µM) against gyrA WT and MTs, surpassing M9, M11, and the control antibiotics. M2 demonstrated a consistent binding affinity across all gyrA single mutations and the T83I_D87N double mutation. MD simulations elucidated the structural stability of M2 in complex with gyrA WT and MTs. The analysis demonstrated consistent RMSD values and minimal alterations in the Rg, with variations of approximately 0.1 nm. RMSF analysis revealed that M2 binding significantly reduces the fluctuations in the QRDR region. Consequently, the simulations revealed sustained hydrogen bonding interactions throughout the observed period, which were found to be instrumental in maintaining the binding affinity between M2 and the gyrA proteins. H-bond occupancy analysis showed that M2 forms stable hydrogen bonds throughout the 100 ns simulation, maintaining key interactions with high occupancy, particularly in all mutant gyrA complexes. The longer residence time and greater persistence of hydrogen bonds in M2-bound complexes suggest stronger target engagement, potentially leading to prolonged enzyme inhibition. The H-bonds were stable with low relative fluctuations, the same has been correlated with overall average low RMSD, which depicts least conformational distortion despite interaction with the M2. The IE profiles revealed favorable binding between M2 and both WT and MT protein variants, demonstrating M2’s efficacy in inhibiting gyrA proteins despite resistance-associated mutations. These findings collectively demonstrate that M2 induces greater structural stability, stronger intermolecular interactions, and reduced flexibility in the QRDR, providing a molecular basis for its enhanced inhibitory potential against fluoroquinolone-resistant *P. aeruginosa*. Binding free energy calculations quantified the thermodynamics of M2 and gyrA WT and MTs interactions. The MM/GBSA and MM/PBSA methods provided estimates of ligand binding affinity by calculating molecular mechanics energy, solvation energy, and optionally entropy. The final binding free energy helped in understanding their interaction strength. Both these methods have shown similar trends in gas-phase energy (GGAS) which is based on VDWALLS and EEL contributions with negligible changes where differ slightly in solvation free energy, split into polar (EGB/EPB) and non-polar (ESURF/ENPOLAR) contributions. The gyrA_WT-M2 complex demonstrated a binding energy of − 22.63 kcal/mol in MM/GBSA and − 14.19 kcal/mol in MM/PBSA, whereas the T83I_MT-M2 complex exhibited the most favorable energy (− 30.82 kcal/mol and − 22.18 kcal/mol respectively). The D87G_MT, D87N_MT, and double mutation T83I_D87N also displayed favorable binding energies of -23.55 kcal/mol, -23.11 kcal/mol, and − 25.06 kcal/mol in MM/GBSA and − 16.36 kcal/mol, – 15.85 kcal/mol and − 15.38 kcal/mol in MM/PBSA respectively. These results highlight M2’s capacity to form strong interactions with WT and mutant proteins. The binding energy of the D87Y_MT-M2 complex was determined to be − 14.02 kcal/mol and − 12.34 kcal/mol based on these two methods, indicating stable but relatively weaker interactions compared to gyrA WT and other mutant proteins. PCA revealed distinct conformational landscapes of gyrA_WT-M2 complexes. The apoprotein gyrA exhibited a broad energy landscape with multiple shallow basins, indicating high conformational flexibility in the absence of a ligand, which is characteristic of unbound proteins exploring a range of metastable states. The PCA-derived free energy landscapes (FELs) indicate that M2-bound gyrA complexes occupy a more restricted conformational space, with lower structural variance and reduced conformational entropy, particularly in mutant gyrA variants (T83I, D87N, D87G, and T83I_D87N). This suggests that M2 stabilizes gyrA in a rigid, less dynamic conformation, potentially impairing its ability to undergo the conformational transitions necessary for DNA supercoiling and catalytic function. This stabilization implies that M2 binding significantly reduces conformational entropy, promoting a compact and stable protein structure. The reduced conformational entropy in M2-bound complexes implies that the ligand may restrict essential enzymatic motions, such as ATP-dependent strand passage and DNA cleavage-reunion cycles, which are critical for bacterial replication^36^. This structural constraint may render fluoroquinolone-resistant *P. aeruginosa* more susceptible to inhibition, as the enzyme’s catalytic function is effectively disrupted. Changes in conformational entropy (flexibility) did not affect the drug-binding due to their high affinity to the active site, as evident from consistent H-bond formations and low interaction energies. The resultant rigidity (reduced conformational entropy) might hinder the protein’s catalytic activity, which further supports the efficiency of the drug candidate M2. These findings provide a biophysical basis for M2’s inhibitory potential, highlighting its ability to stabilize resistant gyrA variants in a non-functional state, thereby overcoming fluoroquinolone resistance. The PCA results also revealed M2’s capacity to stabilize both wild-type and mutant gyrA proteins. Comparative analysis of M2-WT and MTs complexes demonstrated that M2 exhibited efficacy against gyrA_WT and nearly all MT forms, displaying strong binding affinity and dynamic stability. However, destabilizing mutations such as D87G and T83I_D87N may present challenges and potentially reduce M2’s efficacy. The stabilization observed in the T83I_D87N double mutant suggests that M2 can attenuate, but not fully counteract the destabilizing effects of the dual mutations. Subsequent investigations should focus on the structural optimization of M2 compound to enhance its binding affinity and stabilize highly destabilized mutants such as D87G and T83I_D87N.

Ciprofloxacin and Levofloxacin, well-established fluoroquinolone antibiotics, inhibit gyrA by stabilizing the cleavage complex, preventing DNA relegation, and ultimately leading to bacterial cell death^37^. Their pharmacophores comprise a quinolone core essential for enzymatic inhibition, a carboxyl (-COOH) and ketone (C = O) group for Mg²⁺ chelation, and a fluorine (-F) substitution that enhances bacterial penetration^38,39^. In contrast, the quinoline analog N-benzylquinoline-8-sulfonamide (M2) represents a potential novel gyrA inhibitor with a distinct pharmacophoric profile. Lacking the fluoroquinolone’s Mg²⁺-dependent interaction mechanism, M2 instead features a sulfonamide functional group (R − S(= O)_2_ − NR_2_), enabling hydrogen bonding with key active-site residues, and a benzyl moiety, contributing to hydrophobic stabilization within the enzyme pocket^40,41^. Molecular docking and MD simulation data indicate that M2 exhibits comparable binding affinity and stable interaction with gyrA, while its alternative inhibition mechanism suggests potential efficacy against fluoroquinolone-resistant *P. aeruginosa*. Furthermore, unlike CIP and LEV, which are associated with severe adverse effects such as neuropsychiatric toxicity, nephrotoxicity, mutagenicity, seizures, retinal detachment, peripheral neuropathy, aortic aneurysm and aortic dissection, etc^42,43^. M2’s sulfonamide scaffold may offer an improved toxicity profile. Its ability to circumvent fluoroquinolone resistance mechanisms while maintaining stable interactions with gyrA underscores its potential as a novel lead compound, warranting further in vitro and in vivo evaluation to elucidate its therapeutic potential. This study identifies N-benzylquinoline-8-sulfonamide (M2) as a potent inhibitor of gyrA, surpassing ciprofloxacin and levofloxacin in binding affinity and stability. Molecular simulations confirmed its robust interaction with both wild-type and mutant gyrA, indicating its therapeutic potential against fluoroquinolone-resistant *P. aeruginosa.*

## Conclusion

This study identifies quinoline analog N-benzylquinoline-8-sulfonamide (M2) as a potent inhibitor of DNA gyrase subunit A (gyrA WT and MT proteins in fluoroquinolone-resistant *P. aeruginosa*. Molecular docking and free energy calculations (MM/PBSA and MM/GBSA) demonstrated that M2 exhibits more potent and stable binding to both wild-type and mutant gyrA (T83I, D87N, D87Y, D87G, and T83I_D87N) in comparison to ciprofloxacin and levofloxacin, which are standard fluoroquinolone antibiotics. MD simulations elucidated that M2 binding induces structural stabilization of the QRDR region, thereby reducing enzyme flexibility, which may inhibit DNA supercoiling and bacterial replication with greater efficacy than fluoroquinolones. Furthermore, ADMET predictions suggest that M2 has a favorable pharmacokinetic and toxicity profile, avoiding key adverse toxic effects of fluoroquinolones. DFT analysis supports its high chemical reactivity, making it an optimal candidate for further preclinical development. Overall computational analyses demonstrated M2’s superior binding affinity, stability, and interaction energetics compared to standard antibiotics. These findings underscore M2’s potential as a next-generation therapeutic candidate against fluoroquinolone-resistant *P. aeruginosa* to combat the growing threat of antimicrobial resistance. However, further in vitro and in vivo experimental validation is essential to confirm its efficacy and safety, paving the way for its development into a clinically viable treatment. By addressing the limitations posed by complex mutations, M2 could be further developed into a robust antimicrobial candidate capable of combating multidrug-resistant pathogens.

## Methods

### Structural retrieval of target proteins

The target protein DNA gyrase subunit A (gyrA) sequence was retrieved from UniPort [UniProt ID: P48372] and used in the NCBI-BLASTp tool to search for the availability of the crystal 3D structure of the target proteins in the RSCB Protein Data Bank (RSCB-PDB). Because of the unavailability of the respective protein, we employed an extensive protein modeling approach to choose the protein using a homology modeling method based on SWISS-MODEL and GUI-based EasyModeller 4.0 platform^44,45^.

### Modeled protein refinement & validations

The modeled target protein gyrA was refined through the web-based server GalaxyRefine (https://galaxy.seoklab.org/index.html) to improve the quality of the protein structure based on RMSD, molProbity, clash score, poor rotamers and percentage of ramachandran favored^46^. After refinement, the protein was energy-minimized using Swiss-PDB Viewer v4.1.0 (SPDBV) in vacuo with a force field of GROMOS96 43B1 with 2000 steps considering the steepest descent and conjugate gradients^47^. This extensive modeling approach reduces errors in the local conformation and enhances the accuracy of the modeled protein structure. The tertiary structure of the modeled protein was characterized using ProsSA-web, which verified the quality of the local and global model quality^48^. The modeled protein was also validated using ERRAT by assessing the nonbonded interactions between the types of atoms and plots and PROCKECK for stereochemical and overall geometry^49,50^. Finally, the INTERPRO server was employed to elucidate the functional domains within the gyrA protein structure^51^.

### Selection and retrieval of mutants

The target protein gyrA and it’s most important frequently observed single and double mutation information collected from various literature. These mutations were then induced in the respective target proteins through the mutagenesis wizard tool embedded in PyMOL v3.1.1 software.

### Analysis of target protein and its mutants

A structure-based prediction server, DynaMut2 (https://biosig.lab.uq.edu.au/dynamut2/), was employed to assess changes in stability and flexibility due to mutations. This helps in checking protein motion, flexibility analysis, and visualization. DynaMut2 incorporates normal mode analysis (NMA) and graph-based signatures to determine how mutations affect protein stability and dynamics^52^. The generated models were employed to determine the residue cross-correlation. This analysis facilitated the creation of a dynamical cross-correlation map (DCCM), which illustrated the correlated (depicted in red) and anticorrelated (shown in blue) regions within the protein’s structural framework. It determines the correlation between these two residues. If the correlation between two residues was 1.0, the fluctuations of both residues were completely correlated (same period and same phase); if it was − 1.0, the fluctuations of both residues were completely anticorrelated (same period and opposite phase); and if it was 0, the fluctuations of both residues were not correlated. The DCCM plot provided valuable insights into the dynamic behavior of the modeled gyrA protein. The stability of the protein was classified as either stabilizing or destabilizing based on the ΔΔG value, representing the difference in folding free energy change between the mutant and wild-type proteins. A ΔΔG value less than zero indicates a destabilizing mutation, while values exceeding zero signify a stabilizing mutation^53^.

### Protein backbone dynamics and folding free energy determination

The backbone dynamics of specific mutant proteins were evaluated at the residue level using the DynaMine tool, available through the Bio2Byte web interface. This analysis involved the computation of backbone N–H S^2^ order parameter values. The experimental NMR chemical shifts, as represented by these S^2^ values, elucidate the restricted orientations of atomic bond vectors within the context of the molecular reference frame. Notably, S^2^ values exceeding 0.8, signify highly rigid conformations^54^. The web-based server SWOTein was utilized to detect structural stability changes caused by mutations using statistical functions related to folding patterns, local interaction networks, hydrophobic forces, and tertiary interactions. It effectively computed folding free energy values (∆G) for all the residues by utilizing structural descriptors, including solvent exposure, dihedral angles, and alpha-carbon distances^55^.

### Coarse-grained dynamics simulation

The CABS-flex 2.0 server was used for an efficient and rapid modeling approach in wild and mutant protein structure flexibility simulations^56^. It implements an efficient simulation engine that facilitates the modeling of large-scale conformational transitions in protein systems and incorporates the modeling of the loop flexibility and dynamics of selected protein fragments. In the course of the simulations, predefined values were employed to constrain the upper and lower limits for atomic pairs within designated spatial domains. To prevent unstable conformations exceeding predetermined ranges, restraints were implemented. The optimization of coarse dynamics simulations and consensus protein variations in aqueous media, derived from all-atom molecular dynamics simulations (10 ns with suitable force fields), was achieved through the application of these predefined parameters. These default settings incorporated conformational distances of 3.8 and 8.0, along with a constrained gap of 3 (representing the minimal distance between successive amino acids subject to restraint). The root mean square fluctuation (RMSF)values analyzed flexibilities, providing insights into the kinetics of protein and protein-ligand complex interactions.

### Ligand-based analogue screening, pharmacokinetics and antibacterial property prediction

Quinoline, also known as benzopyridine, 1-aza-naphthalene, or chinolin, an aromatic heterocyclic double ring structure-based (fusion of a benzene and a pyridine ring) organic compound, was selected in our study as a reference for screening and assessing analogous compounds due to its similar core structure to fluoroquinolones, which are widely utilized and considered an important drug of choice for treating infections caused by *P. aeruginosa*. The SwissSimilarity server (http://www.swisssimilarity.ch/) facilitated an analog screening process within the integrated two-dimensional and three-dimensional ‘lead-like’ domain of commercial compounds sourced from the ZINC database. This methodology was employed to evaluate compounds, taking into consideration their stereochemical properties, structural alignments, and conformity to pharmacophore models^57^. Using SwissADME, the analogs were evaluated and compared based on their physiochemical properties, violation of lead likeness, pharmacokinetics, medicinal chemistry, and drug-like properties. The specific characteristics examined included gastrointestinal (GI) absorption, blood-brain barrier (BBB) penetration, and synthetic accessibility. Additionally, other crucial bioavailability factors, such as molecular weight (MW), lipophilicity (XLOGP3), polarity (TPSA), solubility (log S), and flexibility, were used to determine the potential of these compounds as effective therapeutic agents against the targets. These parameters illustrate the desirable characteristics of cellular uptake, kidney elimination, ease of synthesis, and oral absorption, which are influenced by the crucial physicochemical attributes^58^. The toxicity parameters of the screened compounds were further evaluated using the ProTox 3.0 server (https://tox.charite.de/protox3/), which defines integrated predicted LD50 values, toxicity class, major toxicity endpoints, that is, hepatotoxicity, nephrotoxicity, cardiotoxicity, carcinogenicity, mutagenicity, and other cellular toxicity factors. Compounds with no toxic effects and pharmacokinetic profiles comparable to those of standard drugs were selected for further study^28^. The antibacterial properties of the final ADMET-screened compounds were evaluated using the PASS online tool (https://www.way2drug.com/passonline/)^59^.

### Structure retrieval of ligands

The PubChem database (https://pubchem.ncbi.nlm.nih.gov/) provides SDF format files for conventional fluoroquinolone antibiotics, that is, ciprofloxacin (CIP) (ID: 2764) and levofloxacin (LEV) (ID: 149096), which were used as control antibiotics^60^. Quinoline analogs utilized in this research underwent ADMET property-based screening and were obtained in SMILES format. 3D structures of the reference antibiotics and screened quinoline analogs were constructed using the OpenBabel software package prior to conducting molecular docking simulations. Throughout this study, the terms ‘ligand’ was used synonymously for quinoline analogues/antibiotics/compounds^61^.

#### Virtual screening through pyrx

The ADMET-screened quinoline analogs were virtually screened using PyRx (python-based GUI v0.8), an open-source virtual screening software for computational drug discovery that was used to screen compounds against drug targets to narrow down the potential ligands^62^. PyRx employs blind docking, an approach in which the entire compound region is selected and the ligand attaches to the enzyme’s catalytic site during docking rather than docking at a specific site. Ligands exhibiting relatively low binding energies were chosen for further analyses. The selected compounds underwent DFT optimization and were subsequently subjected to site-specific molecular docking evaluation.

### Ligand optimization upon quantum chemical DFT simulation

Quantum chemical density functional theory (DFT) simulations facilitate the understanding of the chemical properties, chemical reactivity, and optimization of the structural stability of compounds^63,64^. Quantum chemical calculations were conducted using the Gaussian-09 software to investigate the chemical reactivity and stability of the candidate compounds through DFT simulations^65^. Subsequently, the obtained data were visualized using GaussView version 6.1.1 software^66^. The optimization of molecular geometry, mapping of electron density, and calculations of frontier orbitals (HOMO LUMO) were conducted utilizing a combination of Becke’s three-parameter exchange-correlation function (B3) and Lee-Yang Parr (LYP), in conjunction with the 6–311 + + G(d, p) basis set, to ascertain the lowest energy configurations. The charge distribution was further investigated through the molecular electrostatic potential (MEP), demonstrating electron density transfer. The chemical reactivity and global reactivity descriptors were calculated from FMO. This analysis encompassed the energies of frontier molecular orbitals (FMO) and their corresponding HOMO-LUMO, as well as various other parameters including electron affinity (EA), electronegativity (χ), chemical potential (µ), electrophilicity index (ω), chemical hardness (η), and softness (S)^67^.

### Molecular docking analysis

Molecular docking analysis of the refined proteins with optimized control antibiotics and the best three compounds were performed using the AutoDock 4.2 software^68^. Prior to docking analysis, the gyrA WT and MT protein targets were optimized. During site-specific docking, our major focus was on quinolone-resistance determining regions (QRDRs), which were located between 67 to 106 of gyrA protein^69^. The molecular docking protocol has been derived from previous studies conducted by our research group, which have been successfully validated through in vitro and clinical observations^70–76^. This docking process involved the addition of polar hydrogen atoms, application of Kollman charges, and consolidation of non-polar hydrogen atoms. These steps were implemented to stabilize the free-end residues of the protein structure. Following the application of Gasteiger charges, the torsion of the ligand was fixed. The active-site residue coordinates were employed as the center for constructing an affinity grid box. This box had dimensions of 60 Å^3^ and utilized a uniform spacing of 0.375 Å. The critical active site residues were identified from previous literature and constructed with appropriate dimensions to encompass the entire active site domain. Further validation of the drug-binding pockets located within the active site was conducted utilizing the INTERPRO, CASTp, and DoGSiteScorer servers^77,78^. Grid parameter files (GPF) and docking parameter files (DPF) were generated using AutoDock suite. Employing Lamarckian and genetic algorithms, molecular docking was performed to generate potential binding poses. Molecular docking was performed in triplicate for all the complexes. The protein-ligand complex exhibiting the lowest binding energy (BE) was selected for subsequent analysis in this study. The docked complexes that revealed multiple intermolecular interactions were visualized using Discovery Studio Visualizer v20.1.0.19295. Site-specific molecular docking of gyrA WT and MTs with DFT-optimized M2, M9, and M11 was performed.

### Molecular dynamics simulation

MD simulations were conducted utilizing the GROMACS 2024.2 software to evaluate the stability and dynamic behavior of the docked complexes. The CHARMM36 force field was employed to generate the topologies of the protein, as it is widely recognized for its capacity to accurately model molecular-level interactions and backbone conformations in protein systems^79^.

To neutralize the system, 12 chloride (Cl⁻) ions were introduced into the solvated models of the docked complexes and proteins. The energy minimization step was executed using the steepest descent algorithm with the verlet cutoff method, ensuring structural relaxation at a convergence threshold of 10 kJ/mol. Each energy minimization cycle comprised 50,000 steps to achieve the optimal geometry for the subsequent simulations. The system was equilibrated in two phases: a constant-volume (NVT) ensemble followed by a constant-pressure (NPT) ensemble, each executed for 100 ps. The simulations were conducted under physiological conditions, maintaining a temperature of 300 K and pressure of 1 atm, with a 2 fs integration time step. Subsequent to equilibration, a production molecular dynamics simulation spanning 100 ns was implemented to elucidate the dynamic characteristics of the systems immersed in an explicit aqueous medium. Trajectory analyses were performed to assess the structural and dynamic stability. Several key parameters were evaluated, including root mean square deviation (RMSD), root mean square fluctuation (RMSF), radius of gyration (Rg), hydrogen bonding patterns, solvent accessible surface area (SASA), and Lennard-Jones short-range interaction energy (LJ-SR, representing van der Waals interactions). These metrics provided insights into the conformational behavior, stability, and interaction dynamics of the individual WT protein and all docked complexes throughout the simulation.

### MM/GBSA and MM/PBSA analysis for binding free energy calculation

Binding free energy calculations for the protein-ligand complexes were performed using the gmx_mmpbsa tool, employing the Generalized Born Surface Area (MMGBSA) and Molecular Mechanics Poisson–Boltzmann Surface Area (MMPBSA) methods. These methods validated the molecular dynamics (MD) simulation results and provided a comprehensive evaluation of the binding affinity of ligand M2, which exhibited the highest binding affinity for both the wild-type (WT) gyrA protein and its mutants (MTs). The gmx_MMPBSA test tool was employed to calculate the total binding free energy for each protein-ligand complex based on the molecular dynamics simulation trajectory^80^. This tool computationally calculates binding free energy with GB (Generalized Born) and PB (Poisson-Boltzmann) solvation models. Default settings were employed for all parameters to ensure consistency and reproducibility. The system temperature was maintained at 298.15 K, whereas the solvation parameters ‘igb’ and ‘ipb’ were set to 5 and 2 for MM/GBSA and MM/PBSA, respectively to account for implicit solvent effects. Other key parameters, included internal and external dielectric constants, were set to 1.0 and 78.5 for MM/GBSA, whereas 1.0 and 80.0 were set for the MMPBSA analysis, reflecting standard conditions. The results from the MM/GBSA and MMPBSA analysis provided critical insights into the energetics of ligand binding, elucidating variations in binding free energy between the WT and MT complexes and contributing to the understanding of ligand interaction dynamics. The gmx_MMPBSA_ana visualization tools were employed to graphically represent and assess the energy components of MM/GBSA.

### Principal component analysis

To evaluate the metastable conformational spaces surrounding the native state of WT and MT proteins, Principal Component Analysis (PCA) was employed on molecular dynamics simulation (MDS) trajectories^81^. The consistency of the structural clusters and Free Energy Landscape (FEL) basins derived from PCA parameters validated the accuracy of these conformational spaces. The FEL basins revealed distinct conformational ensembles, indicating differences in equilibrium states between the wild-type and mutated proteins. PCA was conducted utilizing covariance matrices (CM) constructed from the atomic coordinates of protein alpha-carbon (Cα) atoms, thereby minimizing statistical noise. The collective motions of the protein are represented as eigenvectors (direction) and eigenvalues (magnitude), with the initial eigenvectors corresponding to principal components (PCs) capturing the largest eigenvalues. To further refine the analysis, the cosine content of each PC was calculated to determine the degree of harmonicity in protein motion. PCs with cosine content values < 0.2 were selected for interpreting the FEL, ensuring a focus on biologically relevant restrained motions. FEL plots were generated to identify the energetically favorable states of the protein, with minimized structures representing the most stable conformations. The trajectories were projected along the two principal components of highest significance (PC1 and PC2) to construct 2D and 3D free energy landscape (FEL) visualizations, providing insights into the conformational landscapes of both the original and mutant protein-ligand complexes. All PCA and FEL analyses were performed utilizing built-in functions of the GROMACS suite. Covariance matrices were generated and trajectories were projected onto eigenvectors using the ‘gmx covar’ and ‘gmx anaeig’ modules, respectively. FEL plots were created utilizing the ‘g_sham’ module^72^. These analyses facilitated the comparison of dynamic properties and equilibrium states between wild-type and mutant complexes, enhancing the understanding of the structural dynamics underlying their functional differences.

## Electronic supplementary material

Below is the link to the electronic supplementary material.

Supplementary Material 1

Supplementary Material 2

Supplementary Material 3

Supplementary Material 4

## Data Availability

The entirety of the data relevant to this manuscript has been incorporated within the main document or included in the supplementary files provided.
